# Environmental Exposure to Triclosan and Male Fecundity: A Prospective Study in China

**DOI:** 10.3389/fpubh.2022.814927

**Published:** 2022-04-11

**Authors:** Wenting Zhu, Chong Xie, Shasha Zhao, Dan Zhang, Hao Zhang

**Affiliations:** ^1^Key Laboratory of Reproductive Genetics (Ministry of Education), Department of Reproductive Endocrinology, Women's Hospital, Zhejiang University School of Medicine, Hangzhou, China; ^2^Center for Reproductive Medicine, The International Peace Maternity and Child Health Hospital, Shanghai Jiaotong University School of Medicine, Shanghai, China; ^3^Department of Clinical Laboratory, Shanghai Tenth People's Hospital of Tongji University, Shanghai, China; ^4^Key Laboratory of Reproductive Genetics, Zhejiang University, Ministry of Education, Hangzhou, China; ^5^Department of Preventive Dentistry, Shanghai Stomatological Hospital & School of Stomatology, Fudan University, Shanghai, China; ^6^Shanghai Key Laboratory of Craniomaxillofacial Development and Diseases, Fudan University, Shanghai, China

**Keywords:** endocrine disrupting chemical (EDC), triclosan, male fecundity, infertility, prospective study

## Abstract

Triclosan (2,4,4′-trichloro-2′-hydroxy-diphenyl ether, TCS) is widely used in personal care and household products. It is ubiquitous across the ecosystem nowadays. Several *in vitro* and *in* vi*vo* studies have suggested the possible adverse effects of TCS on male reproductive health. However, little research has been done on human beings, especially in eastern countries. To assess the effects of TCS exposure on male fecundity, we recruited couples who planned to conceive and went to the preconception care clinics for physical examination in Shanghai, China. TCS was quantified in male urine samples collected at enrollment. For this study, 443 couples were included in the cohort, and 74.7% of couples (*n* = 331) were prospectively followed 12 months later. The outcomes of interest included the pregnancy status of their wives and time to pregnancy. Elevated male urinary TCS concentrations were found to be associated with diminished fecundability (fecundability odds ratio (FOR) 0.77; 95% CI, 0.62–0.97). The risk of infertility significantly increased (OR = 1.6; 95% CI, 1–2.6) as TCS levels elevated. Besides, we divided TCS concentration into tertiles *a priori*, and there tended to be a dose-response pattern in both analyses. Our findings suggest that environmental exposure to TCS may have an adverse impact on male fecundity.

## Introduction

Triclosan (2,4,4′-trichloro-2′-hydroxy-diphenyl ether, TCS) is a broad spectrum biocide added routinely in a wide array of antiseptic wash products, laundry detergents, cosmetics, and household products (1). Many antimicrobial consumer products are washed down the drain after being used. Despite sewage treatment, a small portion of TCS is still discharged into the environment (2). With the development of the economy, the usage of antiseptic products increases worldwide, and more and more TCS is released into the environment. It is reported that TCS has reached surface water, groundwater, soil, and even drinking water. Moreover, it can accumulate in sediment and aquatic organisms (3).

The uptake of TCS in humans occurs *via* two main routes, namely dermal contact and oral route (ingestion of contaminated drinking water and food) (4, 5). Besides the direct use of TCS products, long-term environmental exposure to TCS also leads to TCS uptake. The majority of TCS is eliminated in the urine in the form of glucuronide conjugate (1).

As it is obviously ubiquitous across the ecosystem, the effects of TCS on human health have attracted attention. Nowadays, TCS has also been detected in diverse human body fluids and tissues (e.g., urine, plasma, milk, and fingernail) (6–9). Research in animals shows that TCS is suspected to be an endocrine-disrupting chemical (EDC) and may adversely affect male sperm quality. *In vitro* study by Kumar et al. (10) demonstrated that TCS disrupted intermediate steroidogenic cascade and depressed testosterone synthesis. Kumar et al. (11) found that TCS decreased the production of androgens and reduced semen production in treated male rats. Lan et al. (12) found TCS induced sperm toxicity and epididymal damage in rats due to the epididymal accumulation tendency of TCS. The study of Bruno GM et al. even indicated maternal (placenta and lactation) exposure to TCS compromised sperm parameters in rats of the F1 generation (13).

Several studies of TCS exposure in men take semen quality as the primary outcome. Many reported a significant statistical association between TCS exposure and semen parameters. Nassan et al. (14) reported lower percent morphologically normal sperm in men with urinary triclosan in the second or third quartile than those undetectable concentrations. The findings of Zhu et al. (15) suggested the adverse effect of TCS on semen quality at the environment-relevant dose. Jurewicz et al. (16) found that triclosan exposure is associated with poorer semen quality. However, the absolute value difference among semen parameters of different TCS exposure groups was moderate, and neither dose-response effect was pronounced (14, 15). Therefore, we wonder whether TCS exposure in men adversely affects male fecundity (his biological capacity to reproduce). The evidence of TCS's potential effects on male fecundity is still limited to date, especially in Asian countries. The current study aimed to fill this void by evaluating the association of urinary concentrations of TCS in men and male fecundity.

## Methods

### Population

This study recruited couples who went to male preconception care clinics in a university affiliated teaching hospital for pre-pregnancy eugenic check from November 2013 to March 2014. Enrolled couples were married (men ≥ 22 years old and women ≥ 20 years old) and planned to conceive recently. Couples who had continuously tried to conceive over 12 months but failed were excluded. All couples signed written informed consent. A trained interviewer obtained information on couples' demographic characteristics, living and working environment, sexual and reproductive status, health-related behaviors, and medical history that may affect reproductive health through a face-to-face interview. The male participants were asked to provide urine and semen samples. The study was approved by the Ethics Committee of Xinhua Hospital Affiliated to Shanghai Jiao Tong University School of Medicine, Shanghai, China (XHEC-C-2013-001).

### Follow-Up

The couples were then prospectively followed *via* a telephone interview after 12 months. Information collected included contraceptive use, pregnancy status, hospital visit (if any), and time to pregnancy (TTP). TTP is an epidemiologic metric widely used for studies of human fecundity (17). TTP was defined as the time length required to achieve spontaneous pregnancy. It was a combination of self-reported months of attempts without contraception usage. If the couples did not conceive with TTP over 12 months, they were labeled as infertile. Fecundability odds ratios (FORs) were calculated to estimate the odds of becoming pregnant each month. A FOR <1 denotes a reduction in fecundity or longer TTP, and a FOR > 1 denotes a shorter TTP.

### Measurement of Urinary Triclosan

A single spot urine sample (100 ml) was collected in a sterile polypropylene cup from each male participant, dispensed into polypropylene tubes (15 ml, 430791 Corning CentriStar), and stored at −80°C until measurement. Total urinary TCS concentration (free and conjugated) was measured using the method reported previously (18). After thawed at 4°C, 10 μl internal standard TCS-D3 (2μg/mL, Dr. Ehrenstorfer GmbH, Augsburg, Germany) was added into a 4 ml urine sample. Firstly, the urine samples were incubated with 20,000 IU/ml β-glucuronidase (Type H-1 from Helix pomatia, Sigma-Aldrich, St. Louis, MO, USA) to hydrolyze the conjugated TCS, then concentrated on a solid-phase extraction column (500 mg/3 ml; Supelco, Bellefonte, PA, USA). Thirdly, the concentrated TCS was vaporized by speed vacuum concentrator and dissolved in 200 μL 90% methanol. The final extract was analyzed by liquid chromatography-electrospray ionization tandem mass spectrometry (Agilent 1290-6490, Agilent Technologies, USA). The limit of detection (LOD) was 0.1 μg/L. Linearity was valid over the range of 0.1–50 ng/ml (*r*^2^ = 0.998). All the intra- and inter-batch precisions were <15%. The recovery was 91.1%. We also measured each urine sample's creatinine level using an enzymatic method on an automatic chemical analyzer (7100 Automatic Analyzer, Hitachi, Japan) to correct the fluctuations of TCS concentrations caused by urine concentration or dilution. Analysts were blinded to all information during the tests.

### Statistical Analysis

All data collected were doubly entered into the EpiData database. Urine TCS values less than the LOD were imputed as LOD/sqrt2 (19, 20). Taking the fluctuations of TCS concentration caused by urine concentration or dilution into consideration, TCS concentration was corrected by dividing the creatinine level of each urine sample. Given that the distribution of TCS concentration was skewed, the corrected TCS concentration was included in the models after natural logarithm transformation.

The student's *t*-test was used for continuous variables with normal distribution and the Wilcoxon rank-sum test for non-normally distributed continuous variables. The chi-square test was used to examine differences in categorical variables. The association between the urinary TCS level and infertility was examined by Logistic regression, while the association between TCS level and fecundability was examined by the Cox model modified for discrete-time data.

Factors that may impact the relationship between TCS exposure and the interest outcomes were considered as confounders (21). Age, smoking status, education, and household income were factors for fecundity and were found to be associated with TCS exposure (22). These confounders were adjusted for in the analysis. Body mass index (BMI) and excessive consumption of alcohol were also risk factors for fecundity (23, 24). These variables were adjusted to improve precision. We also considered male and female reproductive history, given its uncertain relation with fecundity. Although semen quality is an important factor of male fecundity, it was not included in the model to avoid over adjustment (25, 26). Data analysis was performed using SAS version 9.4 (SAS Institute Inc., Cary, NC, USA).

## Results

**Figure 1:**
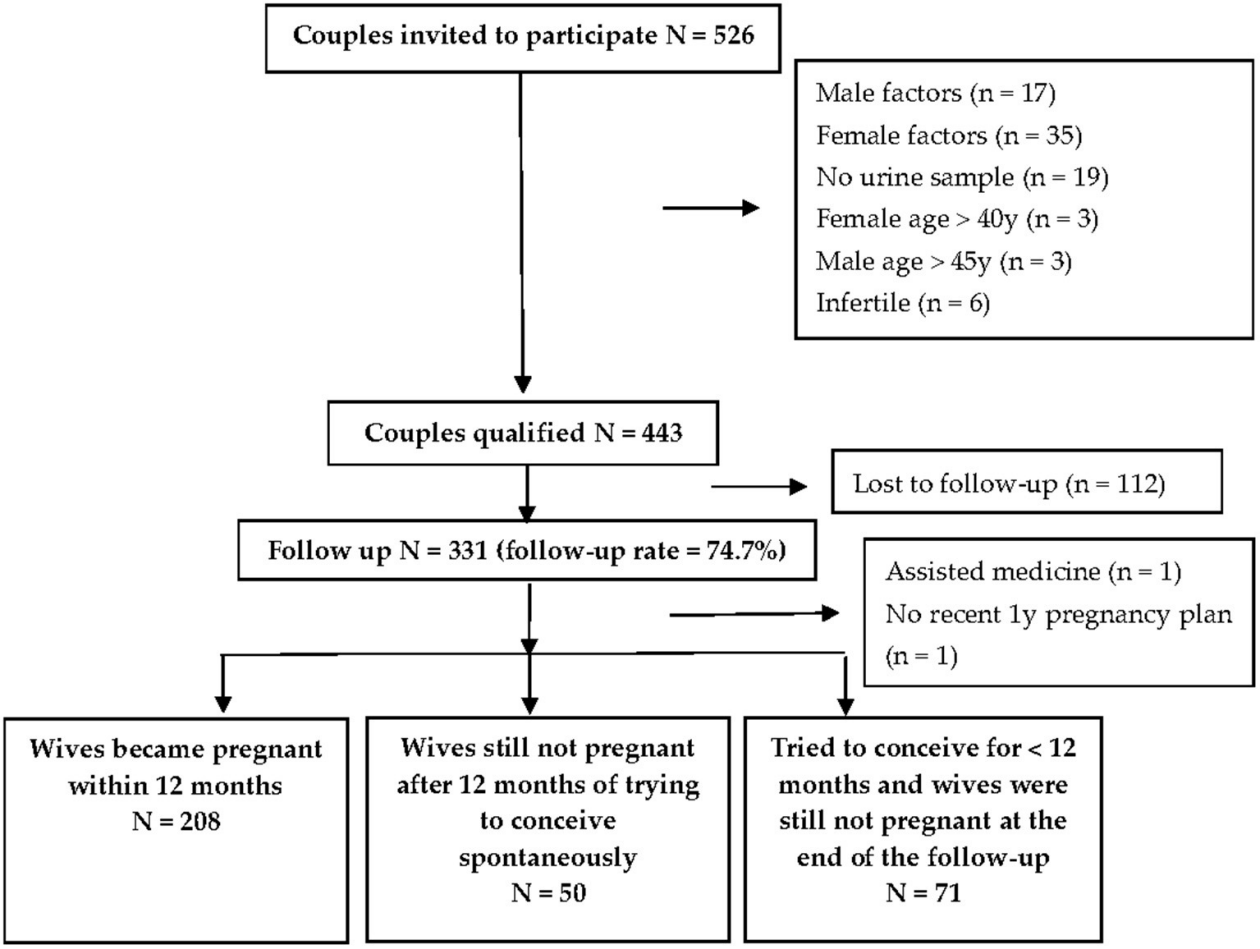
The inclusion and exclusion of participants in the study.

Figure 1 illustrates the inclusion and exclusion criteria of the study population. Among the 526 couples recruited, those with male or female factors that may influence fecundity were excluded. Those with female partners aged 40 years older or male partners aged 45 years older were also excluded, as well as those with male partners who did not provide urine samples or infertile couples (couples who had tried to conceive over 12 months continuously but failed). Male factors included varicocele and cryptorchidism. Female factors included endometriosis, leiomyoma, intrauterine adhesion, uterine malformation, ovarian insufficiency, fallopian tube abnormality, and hyperprolactinemia. Among 443 qualified couples, 96.7% of male urine samples had TCS concentrations above LOD. The median (25th and 75th) of TCS exposure levels in our study was 1.12 (0.5, 3.48) ng/ml. The geometric mean (GM) (25th and 75th) of TCS exposure levels was 1.4 (1.31, 1.62) ng/ml. In total, 331 couples were prospectively followed after 12 months (follow-up rate = 74.7%). Two other couples were also excluded for data analysis, whereas one conceived with the help of medical assistance and the other delayed their pregnancy plan until 2 years later. Among the remaining 329 couples, 208 (63.2%) wives became pregnant within 12 months, 50 (15.2%) wives did not conceive spontaneously despite continuous attempts for 12 months, and 71 (21.6%) wives were still not pregnant at the end of the follow-up since they tried to conceive <12 months.

The baseline characteristics were comparable among pregnant, infertile, and not pregnant couples, except for male reproductive history and corrected TCS concentration (Table 1). Compared with the pregnant group, the men in the infertile group seemed less likely to make their partners pregnant before (66 vs. 42.3%), and corrected TCS concentrations were significantly higher in the infertile group (median 1.29 vs.0.89 ng/mg creatinine). The baseline characteristics of the participants who completed following-up or missing subjects are shown in Supplementary Table 1. Among the missing couples, male partners are relatively older and are less likely to answer family income, while the female partners have fewer college education opportunities and are less likely to get pregnant before.

**Table 1:** The baseline characteristics of couples who are pregnant, infertile, and not pregnant in this study.

**Baseline characteristics**	**Pregnant**	**Infertile**	**Not pregnant**	***P* value**
	***N* = 208**	***N* = 50**	***N* = 71**	
**Male**				
Age (years, mean ± SD)	30.3 ± 3.3	30.3 ± 3.3	30.4 ± 3.6	0.34
BMI (Kg/m^2^, mean ± SD)	24.0 ± 3.1	24.1 ± 3.3	23.9 ± 3.2	0.83
Education (years), *n* (%)				0.08
<16	49 (23.6)	13 (26.0)	26 (36.6)	
16	106 (51.0)	25 (50.0)	37 (52.1)	
>16	53 (25.5)	12 (24.0)	8 (11.3)	
Drinking alcohol ^a^, *n* (%)				0.08
Never	69 (33.2)	14 (28.0)	28 (39.4)	
Seldom	123 (59.1)	35 (70.0)	42 (59.2)	
Frequent	16 (7.7)	1 (2.0)	1 (1.4)	
Smoking, *n* (%)	40 (19.2)	11 (22.0)	22 (31.0)	0.12
Male reproductive history ^b^, *n* (%)				0.003
Never made pregnant	88 (42.3)	33 (66.0)	26 (36.6)	
Previous pregnancy	120 (57.7)	17 (34.0)	45 (63.4)	
Household income (1,000/year), *n* (%)				0.54
<100	71 (34.1)	17 (34.0)	31 (43.7)	
100–300	54 (26.0)	11 (22.0)	14 (19.7)	
>300	73 (35.1)	21 (42.0)	22 (31.0)	
Refuse to answer	10 (4.8)	1 (2.0)	4 (5.6)	
Triclosan, Median (25th, 75th) (ng/ml)	1.11 (0.41, 3.40)	1.57 (0.71, 5.74)	0.90 (0.45, 2.57)	0.080
Corrected Triclosan ^c^, Median (25th, 75th) (ng/mg creatinine)	0.89 (0.38, 2.41)	1.29 (0.71, 6.49)	0.94 (0.41, 2.41)	0.045
**Female**				
Age (years, mean ± SD)	28.1 ± 2.9	28.8 ± 2.9	28.5 ± 3.0	0.31
BMI (Kg/m^2^, mean ± SD)	20.1 ± 2.0	20.0 ± 2.2	20.9 ± 3.0	0.18
Education (years), *n* (%)				0.37
<16	76 (36.5)	12 (24.0)	29 (40.8)	
16	96 (46.2)	28 (56.0)	34 (47.9)	
>16	36 (17.3)	10 (20.0)	8 (11.3)	
Female reproductive history, *n* (%)				0.22
Never got pregnant	123 (59.1)	32 (64.0)	35 (49.3)	
Previous pregnancy	85 (40.9)	18 (36.0)	36 (50.7)	

a *Drinking status definition: Never (<1/month), Seldom (≥1/month, <1/week), Frequent (≥1/week)*.

b *Male reproductive history: Made his wife or ex-girlfriend pregnant or not*.

c *Triclosan concentration was corrected by dividing the creatinine level of each urine sample*.

Table 2 presents the results of the Cox model modified for discrete-time data for the association between TCS concentrations and male fecundability. The remaining 329 couples were all included in the Cox model. Since couples' ages were correlated (*r* = 0.66), only male ages were adjusted in the following analyses. Higher TCS concentrations were positively associated with reduction in fecundability (adjusted OR = 0.77; 95% CI [0.62, 0.97]). Moreover, a significant (*P* = 0.02) trend was observed when we divided the TCS concentrations into tertiles.

**Table 2:** Fecundability odds ratios for triclosan exposure using the Cox model modified for discrete time data.

**Triclosan^**a**^ (ng/mg creatinine)**	***N***	**Unadjusted-OR (95% CI)**	**Adjusted-OR^**b**^ (95% CI)**
**Continuous**	329	0.79 (0.63, 0.98)	0.77 (0.62, 0.97)
**Tertiles**			
<0.56	110	Ref	Ref
0.56–1.8	110	(0.56, 1.2)	0.86 (0.59, 1.2)
>1.8	109	0.69 (0.48, 0.98)	0.65 (0.45, 0.94)
*P* for trend		0.04	0.02

a *Ln-transformed of corrected troclosan concentrations (triclosan concentration was corrected by dividing creatinine level of each urine sample)*.

b *Adjusted for male age, BMI, education, income, current smoking, drinking, male and female reproductive history*.

Multivariable logistic regression models were used to study the effect of pre-pregnancy urinary concentrations of TCS on male infertility. Among the remaining 329 couples, 71 tried to conceive for <12 months and the wives were still not pregnant at the end of the follow-up. Therefore, they were excluded from this analysis. As shown in Table 3, increased TCS concentrations were associated with a higher rate of infertility (adjusted OR = 1.6; 95% CI [1, 2.6]). Furthermore, we divided the TCS concentrations into tertiles. Compared with the lowest tertile (<0.57 ng/mg creatinine), the middle TCS levels (0.57–1.8 ng/mg creatinine) were nearly significantly associated with increased infertility, while the highest TCS levels (> 1.8 ng/mg creatinine) were significantly associated with increased infertility. Moreover, there seemed to be a dose-response pattern (*P* = 0.06).

**Table 3:** The association between triclosan and infertility using a multivariable logistic regression model.

**Triclosan^**a**^ (ng/mg creatinine)**	***N***	**Unadjusted-OR (95% CI)**	**Adjusted-OR^**b**^ (95% CI)**
**Continuous**	258	1.6 (1.1, 2.5)	1.6 (1.0, 2.6)
**Tertiles**			
<0.57	86	Ref	Ref
0.57–1.8	86	2.6 (1.1, 6.1)	2.4 (0.99, 5.9)
>1.8	86	2.8 (1.2, 6.5)	2.5 (1.0, 6.0)
P for trend		0.04	0.06

a *Ln-transformed of corrected triclosan concentrations (triclosan concentration was corrected by dividing creatinine level of each urine sample)*.

b *Adjusted for male age, BMI, education, income, current smoking, drinking, male and female reproductive history*.

## Discussion

We found a positive association between TCS concentration and infertility as well as a negative association between TCS concentration and fecundability in this study. The findings suggest that environmental exposure to TCS may have an adverse impact on male fecundity.

Male fecundity refers to the male component in the biological ability for reproduction, which could be evaluated by the time it takes for the female partner to conceive (27). To our best knowledge, this is the first report showing a negative association between male TCS exposure and TTP.

Recently, TCS has been suspected to be a potential reproductive toxicant. The reproductive endocrine-disrupting effects of TCS were demonstrated *in vivo* and *in vitro* studies through three perspectives. Firstly, TCS was revealed to bind to estrogen and androgen receptors exhibiting estrogen, and anti-androgenic activities (28–31). Secondly, with a chemical structure similar to thyroid hormones, TCS has been shown to disrupt thyroid hormone homeostasis. Finally, TCS may also suppress testicular steroidogenesis and adversely affect male reproduction (32).

The biological mechanism of the impact of TCS on male fecundity is unclear yet. Nevertheless, *in vivo* studies have demonstrated that TCS has anti-androgenic properties and could adversely affect male reproduction and fertility. Ha et al. (32) gave male Sprague-Dawley Rats TCS daily by oral gavage for 31 days and found the inhibition of testicular steroidogenesis through the miR-6321/Map3k1-regulated JNK/c-Jun/Nur77 cascade. Ena et al. (33) found that daily sperm production was significantly diminished with marked inhibition of androgen receptor protein expression with subchronic exposure to high doses of TCS (750 mg/kg) in prepubertal male rats. Subchronic treatment with TCS in weanling male rats was also showed significantly decreased testosterone, luteinizing hormone, follicle-stimulating hormone levels, and a state of testicular oxidative stress, which play a role in testicular DNA damage and endocrine disruption (34). Furthermore, some other cell-based assays revealed that TCS bound to estrogen and androgen receptors exhibit estrogen, and anti-androgenic activities (29, 35). With a similar chemical structure of TCS to thyroid hormones, TCS has been shown to disrupt thyroid hormone homeostasis (36), which is essential for maintaining male reproduction (37).

These findings suggest that TCS may disrupt hormone homeostasis, reduce semen quality, and then affect fecundity in humans. However, population studies on this topic are still in the preliminary stage due to a lack of research. A case-control study conducted by Chen et al. (38) in China found no relationship between TCS exposure and idiopathic male infertility. In another cohort study, Smarr et al. (39) prospectively assessed couples' urinary concentrations of TCS in the context of fecundity, measured as TTP. Nevertheless, no associations were observed when TCS concentrations were modeled continuously. In our previous study, TCS exposure was related to a decrease in sperm concentration, sperm count, the number of forward-moving sperms, and the number of normally morphologic sperms (15). The results are in agreement with the study performed by Jurewicz et al. (16). These findings suggest TCS may be a risk factor of impaired male fecundity to some extent.

Triclosan exposure levels (GM = 1.4 ng/ml) in our study seem to be similar to Asian levels (GM = 1.29 ng/ml) in Japan; GM = 0.99 ng/ml in Korea (40), but lower than American levels (GM = 12.3 μg/L) in Canada (22); GM = 13μg/L in US (7) and European levels (GM = 2–3 μg/L) in Belgium (41); GM = 6.1 μg/L in Spain (42). This may be due to different lifestyles and products used between the western and eastern countries.

Several limitations of our study need to be kept in mind. First, due to the variability of TCS levels in urine, a spot urine sample may not be representative of the usual environmental exposure level of an individual. However, it was reported that TCS concentration in a single urine sample could represent the six-month average exposure to TCS in children 6–10 years old (43). This finding cannot be generalized to adults directly, but adults usually have much more stable environmental exposure with a comparatively stable habitual lifestyle and living environment. Bodin et al. (44) reported that most men did not adjust their lifestyles in the preparation period for pregnancy. Besides, Lassen et al. (45) found modest consistency in repeated measurements of TCS in spot urine samples (intra-class correlation coefficients (ICC):0.55–0.9). Second, we collected self-reported data on TTP, which is subject to recall bias. Nevertheless, the degree of error might be more minor in China. Under the long-term influence of the one-child family policy, couples usually pay much more attention to the process of trying to conceive. Furthermore, since none of the men were aware of their TCS levels, differential misclassification of infertility by TCS levels is unlikely in our study. Third, we did not take TCS exposure in women into consideration in this study. As a successful pregnancy is a couple-dependent outcome, it is possible that TCS affects female fecundity and results in a long TTP and even infertility (18). Couples' TCS exposure was found significantly correlated but modest (*r* = 0.31) (39), which might not be able to explain the total association between reduced male fecundity and TCS exposure. Last but not least, information on the frequency of intercourse was not collected in this study. Infrequent intercourse or intercourse outside of the fertile window can also result in prolonged TTP or infertility. However, these couples who desired to achieve natural conception were given pre-pregnancy eugenic check and preconception education at the reproductive clinic. Thus, we do not think the frequency of intercourse should be a vital issue for the vast majority of couples recruited.

Our prospective study suggests that environmental exposure to TCS adversely impacts male fecundity. But in light of the limitations of this work, our results await corroboration by more large-scale prospective cohort studies of repeated preconception urinary measures of couple's TCS exposure in the context of couple fecundity.

## Data Availability Statement

The raw data supporting the conclusions of this article will be made available by the authors, without undue reservation.

## Ethics Statement

The studies involving human participants were reviewed and approved by the Ethics Committee of Xinhua Hospital Affiliated to Shanghai Jiao Tong University School of Medicine, Shanghai, China (XHEC-C-2013-001). The patients/participants provided their written informed consent to participate in this study.

## Author Contributions

WZ designed the study, analyzed and interpreted the clinical data, and drafted the manuscript. CX collected clinical data and managed the database. SZ measured the level of TCS creatinine concentration in urine. DZ and HZ supervised the project and critically revised the manuscript. All authors read and approved the final manuscript.

## Funding

This study was supported by the National Natural Science Foundation of China (82003470 and 81401253), the China Postdoctoral Science Foundation (2012M520910), Projects of Shanghai Municipal Health Commission (201840140), and Projects of Shanghai Stomatological Hospital (SSDCE-2016-02).

## Conflict of Interest

The authors declare that the research was conducted in the absence of any commercial or financial relationships that could be construed as a potential conflict of interest.

## Publisher's Note

All claims expressed in this article are solely those of the authors and do not necessarily represent those of their affiliated organizations, or those of the publisher, the editors and the reviewers. Any product that may be evaluated in this article, or claim that may be made by its manufacturer, is not guaranteed or endorsed by the publisher.
