# Supplementary material for: Environmental Exposure to Triclosan and Male Fecundity: A Prospective Study in China
Source: Front Public Health. 2022 Apr 11;10:814927. doi: 10.3389/fpubh.2022.814927 (PMC9035866; doi:10.3389/fpubh.2022.814927)
Supplement: Supplementary file 1 [file Table_1.DOCX]

Supplementary Material

# Supplementary Tables

**Supplementary Table 1.** The baseline characteristics of followed and missing couples in this study.

| **Baseline characteristics** | **Follow-up** | **Missing** | **P value** |
| --- | --- | --- | --- |
|  | **N = 329** | **N = 112** |  |
| **Male** |  |  |  |
| Age (years, mean ± SD) | 30.3 ± 3.5 | 31.5 ± 4.3 | 0.01 |
| BMI (Kg/m^2^, mean ± SD) | 24.0 ± 3.2 | 24.0 ± 3.1 | 0.86 |
| Education (years), n (%) |  |  | 0.94 |
| <16 | 88 (26.8) | 31 (27.7) |  |
| 16 | 168 (51.1) | 55 (49.1) |  |
| >16 | 73 (22.2) | 26 (23.2) |  |
| Drinking alcohol ^a^, n (%) |  |  | 0.98 |
| Never | 111 (33.7) | 39 (34.8) |  |
| Seldom | 200 (60.8) | 67 (59.8) |  |
| Frequent | 18 (5.5) | 6 (5.4) |  |
| Smoking, n (%) | 73 (22.2) | 30 (26.8) | 0.32 |
| Male reproductive history ^b^, n (%) |  |  | 0.62 |
| Never made pregnant | 147 (44.7) | 47 (42.0) |  |
| Previous pregnancy | 182 (55.3) | 65 (58.0) |  |
| Household income (￥1,000/y), n (%) |  |  | 0.004 |
| <100 | 119 (36.2) | 32 (28.6) |  |
| 100-300 | 79 (24.0) | 20 (17.8) |  |
| >300 | 116 (35.3) | 44 (39.3) |  |
| Refuse to answer | 15 (4.5) | 16 (14.3) |  |
| Triclosan , Median(25th, 75th) (ng/mL) | 1.11 (0.47, 3.38) | 1.08 (0.50, 4.25) | 0.53 |
| Corrected Triclosan ^c^, Median(25th, 75th) (ng/mg creatinine) | 0.94 (0.43, 2.84) | 1.07 (0.41, 2.98) | 0.93 |
| **Female** |  |  |  |
| Age (years, mean ± SD) | 29.4 ± 2.8 | 29.9 ± 2.4 | 0.09 |
| BMI (Kg/m^2^, mean ± SD) | 20.3 ± 2.3 | 21.2 ± 3.6 | 0.11 |
| Education (years), n (%) |  |  | <0.0001 |
| <16 | 117 (35.6) | 86 (76.8) |  |
| 16 | 158 (48.0) | 14 (12.5) |  |
| >16 | 54 (16.4) | 12 (10.7) |  |
| Female reproductive history, n (%) |  |  | <0.0001 |
| Never got pregnant | 190 (57.8) | 105 (93.8) |  |
| Previous pregnancy | 139 (42.3) | 7 (6.3) |  |

^a^ Drinking status definition: Never (<1/month), Seldom (≥1/month, <1/week), Frequent (≥1/week).

^b^ Male reproductive history: Made his wife or ex-girlfriend pregnant previously or not.

^c^ Triclosan concentration was corrected by dividing creatinine level of each urine sample.
